# Highly Sensitive and Tunable Plasmonic Sensor Based on a Nanoring Resonator with Silver Nanorods

**DOI:** 10.3390/nano10071399

**Published:** 2020-07-18

**Authors:** Chung-Ting Chou Chao, Yuan-Fong Chou Chau, Hung Ji Huang, N. T. R. N. Kumara, Muhammad Raziq Rahimi Kooh, Chee Ming Lim, Hai-Pang Chiang

**Affiliations:** 1 Department of Optoelectronics and Materials Technology, National Taiwan Ocean University, Keelung 20224, Taiwan; suyang191@gmail.com; 2 Centre for Advanced Material and Energy Sciences, Universiti Brunei Darussalam, Tungku Link, Gadong BE1410, Brunei Darussalam; roshan.kumara@ubd.edu.bn (N.T.R.N.K.); chernyuan@hotmail.com (M.R.R.K.); cheeming.lim@ubd.edu.bn (C.M.L.); 3 Taiwan Instrument Research Institute, National Applied Research Laboratories, Hsinchu 300, Taiwan; hjhuang@narlabs.org.tw; 4 Institute of Physics, Academia Sinica, Taipei 115, Taiwan

**Keywords:** plasmonic refractive index sensor, metal-insulator-metal waveguide, figure of merit, sensitivity, nanoring

## Abstract

We numerically and theoretically investigate a highly sensitive and tunable plasmonic refractive index sensor that is composed of a metal-insulator-metal waveguide with a side-coupled nanoring, containing silver nanorods using the finite element method. Results reveal that the presence of silver nanorods in the nanoring has a significant impact on sensitivity and tunability performance. It gives a flexible way to tune the system response in the proposed structure. Our designed sensor has a sensitivity of 2080 nm/RIU (RIU is the refractive index unit) along with a figure of merit and a quality factor of 29.92 and 29.67, respectively. The adequate refractive index sensitivity can increase by adding the silver nanorods in a nanoring, which can induce new surface plasmon polaritons (SPPs) modes that cannot be found by a regular nanoring. For a practical application, a valid introduction of silver nanorods in the nanoring can dramatically reduce the dimension of the proposed structure without sacrificing performance.

## 1. Introduction

Surface plasmon polariton (SPP) is a phenomenon of electromagnetic (EM) waves that propagate along with a dielectric-metal interface [1,2,3,4,5,6]. It can overcome the conventional optical diffraction limit and achieve the transmission and manipulating of the optical signal within the subwavelength scale [7,8]. Plasmonic metal-insulator-metal (MIM) waveguides based on SPPs as a potential branch of optical waveguides are extensively investigated due to their easy on-chip integration, low bent loss, long propagation length, deep subwavelength confinement, and reasonably simple manufacture [9,10,11,12]. Plasmonic refractive index sensors based on MIM waveguides [13,14,15] have attracted numerous considerations because of the requirement for ultrahigh-sensitivity biochemical sensors [16,17]. One of the remarkable aspects of plasmonic MIM waveguides is the side-coupled cavities plasmonic structure operating in the range of visible and near-infrared regions. They have promising applications in optic communications [18], light trapping [19], sensing [20,21], slow light devices [22], and other nanophotonic devices [23,24,25,26].

Recently, different patterns of lateral cavity-coupling based MIM waveguides have been proposed and demonstrated in an experiment and theory [27,28,29]. These SPPs sensors contain a MIM straight waveguide coupled with lateral cavities, such as a gear-shaped nanocavity [30], a T-shaped slot [31], two rectangular resonators [32], and multiple cavities with compound structures [33], etc. The general side-coupled resonators can be circular, triangular, square, disk, hexagonal, and other particular outlines. However, these SPPs sensors currently show low sensitivity and manufacturing difficulty, which is a great challenge to the researchers. It is well-known that a structure with only one resonant mode (e.g., transmittance dip/peak) scarcely expects to have workable applications. However, multiple resonance dips/peaks commonly hint at more complicated compositions leading to difficulties in achieving a high tunability. It is also a technical challenge to size reduction while also assuring high sensing performance in the light of the evolution of high sensitivity requirements.

In this paper, we designed a highly sensitive and tunable plasmonic band-stop filter based on a MIM waveguide-coupled nanoring containing silver nanorods. The proposed structure is variable in the visible and infrared region, and the nanoring size is kept intact. The effective refractive index can be manipulated and changed by introducing the silver nanorods in a nanoring, which can induce new SPPs modes that cannot be found in a normal nanoring. It is worth noting that the sensing performance of the proposed plasmonic sensor is superior to those reported in the literature. Simulation results reveal that the existence of silver nanorods in the nanoring has a significant impact on the sensitivity performance, which is 1.86 times higher than that of the same case without silver nanorods. The refractive index sensitivity can reach up to 2080 nm/RIU (where RIU stands for the refractive index unit), which are remarkably higher than those of SPP-based sensors reported in the literature (e.g., [34,35,36,37,38,39,40]). An underlying physical mechanism of the observed transmittance and propagation properties is illustrated by analyzing spatial distributions of the magnetic field, electric field, and power flow at resonant wavelengths. Moreover, the refractive index sensitivity, along with the figure of merit and quality factor, is calculated. The impact of the geometric parameters corresponding to the transmittance properties has been studied as well. The sensing properties of the proposed band-stop filter can serve as a promising nanosensor. It could provide the role for the realization of highly integrated circuits for constructing plasmonic sensors with a nanoring, containing the silver nanorods for sensing applications.

## 2. Structure Design and Simulation Method

**Figure 1:**
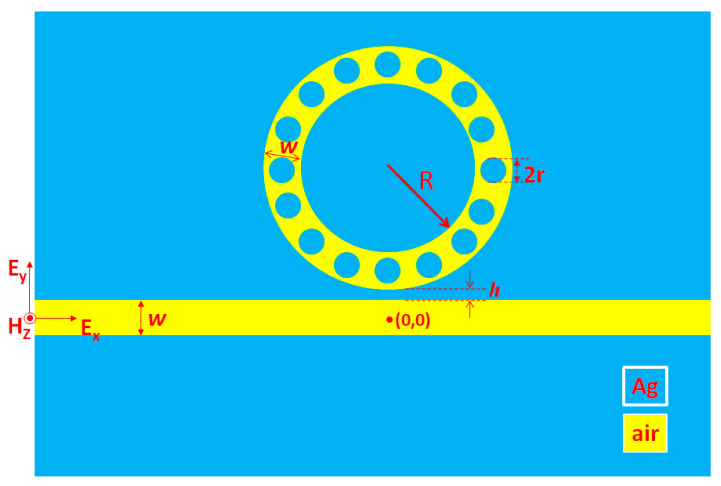
Schematic diagram of the proposed plasmonic band-stop filter, which is composed of a straight waveguide (width *w*), a laterally coupled nanoring (width *w* and core radius *R*) including sixteen silver nanorods in the nanoring. The silver nanorods (radius *r*) uniformly distribute in the nanoring. The gap between the nanoring and the straight waveguide is *h*. The origin point (0,0) of the simulation model is located at the middle-end of the straight waveguide.

Figure 1 shows the simulation model of the proposed plasmonic band-stop filter, which includes a straight waveguide (width *w*); and a laterally coupled nanoring (width *w* and core radius *R*) consisting of sixteen silver nanorods in the nanoring. The silver nanorods (radius *r*) uniformly disperse in the nanoring. The gap-coupled distance between the nanoring and the straight waveguide is *h*. In the schematic diagram, the yellow and cyan colors stand for air and silver, respectively. In real situations, the incident light can propagate into the proposed plasmonic band-stop filter using the photonic crystal fiber (PCF) [41,42] or a dielectric waveguide with a radical grating coupler and a mode-matching technique [43]. Then, the output signal is analogously delivered to a spectrometer and a computer for data analysis. Thanks to the rapid advances in the fabrication technique of nanophotonic structures, the fabrication of the proposed plasmonic MIM waveguide is feasible with the current technology [44,45,46]. A similar structure of a MIM waveguide with a side-coupled nanoring has previously been fabricated by using the physical vapor deposition and a focused ion beam to etch the nanoring [45]. Another fabrication method of a plasmonic filter with a resonator has also been proposed in [46], in which a laser design based on a circular nanoring is covered with metal.

We calculated the transmittance spectrum and sensor sensitivity of the proposed band-stop filter employing a two-dimensional (2D) finite element method (FEM) [47] with perfect matching layer-absorbing boundary conditions along the *x*-axis. A three-dimensional (3D) simulation is reduced to a 2D one, because both models will obtain similar results in simulations [20,21,22] and also the experimental results [48,49]. In addition, the 2D simulation model could save the time and computer resource compared to the 3D one. According to our simulations, the minimum thickness of the structure in the third dimension (i.e., *z*-axis) should be 1.6 μm to obtain approximately similar results as those of the 2D one and a good agreement between the 2D and 3D simulation results. A TM-polarized incident EM wave with an in-plane electric field component, along with the x-direction is directly coupled to the straight waveguide [50]. For symmetry, the origin point (0,0) of the simulation model is located at the middle-end of the straight waveguide. The incident wavelengths are covered 500–2600 nm in the step of 1 nm, and the definition of transmittance (*T*) is *T* = (S_21_)^2^, where *S_21_* is the transmission coefficient.

The complex relative permittivity of silver is from Reference [51]. If the nanoring’s plasmon resonance condition is contented, the resonance wavelength (λ_res_) based on the standing wave theory can be expressed by [52,53]:

(1) $$\lambda_{\mathrm{res}}=\frac{2LRe(n_{\mathrm{eff}})}{m-\frac{\varphi }{2\pi }}\;\left(m=1,2,3\ldots \right)$$

where *L* is the perimeter of the nanoring, and *Re*(*n*_eff_) represents the real part of the effective refractive index of the SPP. *m* is the mode number (positive number, i.e., *m* = 1, 2, 3, …), and *φ* is the phase shift of SPP reflection in the straight waveguide and nanoring, respectively. The phase shift is due to the reflection from the nanoring. The TM mode equation for a MIM waveguide can be expressed as [53]:

(2) $$\tanh\left(k\omega \right)=\frac{-2k{p\alpha }_{c}}{k^{2}+p^{2}\alpha_{c}^{2}}$$

where *k* is the wave vector, *p* = ε_d_/ε_m_ and α_c_ = [*k*_0_^2^(*ε*_d_ − *ε*_m_) + *k*^2^]^1/2^, *ε*_d_ and *ε*_m_ are the permittivity of the dielectric and metal, respectively. *k*_0_ = 2π/λ is the wave vector of the incident light. Therefore, k can solve it from Equation (2). The real part of the effective refractive index *Re*(*n*_eff_) of the MIM waveguide as a function of wavelength can be solved from *n*_eff_ = *k*/*k*_0_ = [*ε*_m_ + (*k*/*k*_0_)^2^]^1/2^.

Moreover, the sensitivity (*S*) can be calculated as S = Δλ/Δn, where Δλ is the shift of λ_res_, λ_res_ is the wavelength at the transmittance dip, and Δ*n* is the difference of the refractive index. In addition, FOM is S/FWHM, where FWHM is the full width at half-maximum of the transmittance spectrum. An important factor of a wavelength filter is the capacity to give high wavelength selectivity, which represents a high-quality factor (*Q*). The quality factor can be defined as Q = λ_res_/FWHM.

## 3. Results and Discussion

**Figure 2:**
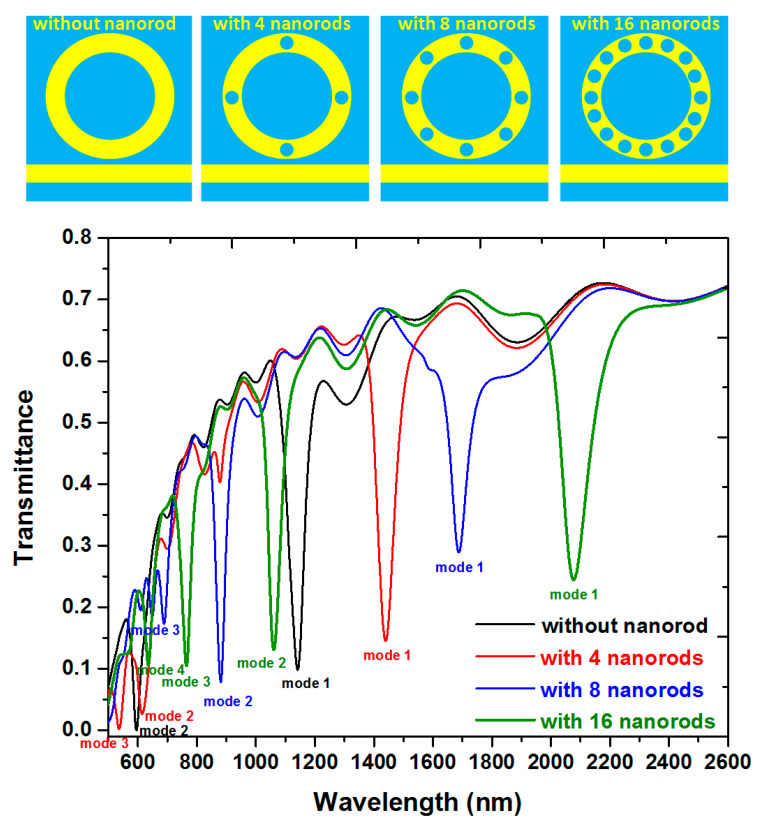
Transmittance spectra of the proposed plasmonic band-stop filter without (black color) and with (red, blue, and olive colors) silver nanorods in the nanoring. The numbers of the silver nanorods are *N* = 0, 4, 8, and 16, respectively, as shown in the most upper panel of this figure. The other geometric parameters, *w*, *h*, *R*, and *r*, are set to be 50, 10, 100, and 20 nm, respectively.

According to Equation (1), the structure parameters (i.e., *R*, *r* and *N*) have a great influence on the resonant condition (i.e., λ_res_, *L* and *n*_eff_) and sensing performance of the proposed plasmonic sensor. For compacting the nanoring’s size, the core radius is set as *R* = 100 nm throughout this paper. To avoid manufacturing complexity, the maximum number of silver nanorods and the radius of silver nanorods are set as *N* = 16 and *r* = 24 nm in the following simulations. The SPPs wave from the nanoring waveguide, directly coupled to the MIM straight waveguide, can be treated as an oscillator. We inspect the transmittance spectra of the proposed band-stop filters without and with silver nanorods in the nanoring, as depicted in Figure 2. The numbers of the silver nanorods are *N* = 0, 4, 8, and 16, respectively, for comparison, as shown in the most upper panel of Figure 2. The other geometric parameters, *w*, *h*, *R*, and *r*, are set to be 50, 10, 100, and 20 nm, respectively. We keep the MIM straight waveguide and nanoring width *w* = 50 nm to guarantee that only the fundamental transverse magnetic (TM_0_) mode can be supported [54]. As shown in Figure 2, the optical performance can easily be manipulated by varying the *N* value of the structure. The transmittance dips, as shown in Figure 2, results from the narrow discrete state (nanoring) and the continuum (straight waveguide). There are two clear resonance dips that occurred at λ_res_ = 1142 and 595 nm (marked by mode 1 and mode 2) with the corresponding Q factor of 25.38 and 29.75, respectively, for the case without silver nanorods (black line), three dips at λ_res_ = 1140, 615, and 536 nm (marked by mode 1, mode 2, and mode 3) with the corresponding Q factor of 28.8, 30.75, and 17.87, respectively, for the case with *N* = 4 (red line), three dips at λ_res_ = 1168, 881, and 689 nm (marked by mode 1, mode 2, and mode 3) with the corresponding Q factor of 42.2, 30.52, and 34.45, respectively, for the case with *N* = 8 (blue line), and four dips at λ_res_ = 2077, 1061, 765, and 636 nm (marked by mode 1, mode 2, mode 3, and mode 4) with the corresponding Q factor of 29.67, 30.31, 38.25, and 30.75, respectively for the case with *N* = 16 (olive lines). These results are much remarkable compared with the previous designs. It can be observed from Equation (1) that this has a linear relationship with the *n*_eff_. The resonance modes found in the cases with silver nanorods are due to the presence of the silver nanorods that will raise n_eff_ in the nanoring. It can also be found that λ_res_ is closely associated with L (including the perimeter of the silver nanorods and the nanoring) of the nanoring resonator. More numbers (*N*) of silver nanorods set in the nanoring will result in more significant L and *n*_eff_ (see Equation (1)), and this will contribute to the increase in the λ_res_. The stopped-wavelength increases steadily with increasing the number of silver nanorods (*N*) set in the nanoring in broad transmittance spectra. It implies a method to manipulate the resonance condition in the nanoring by varying the effective refractive index (*n*_eff_). It can be realized by embedding the different number of silver nanorods into the nanoring. Therefore, one can implement the narrowband filtering function at specific wavelengths by suitably varying *n*_eff_ in the nanoring and incident EM wave.

**Figure 3:**
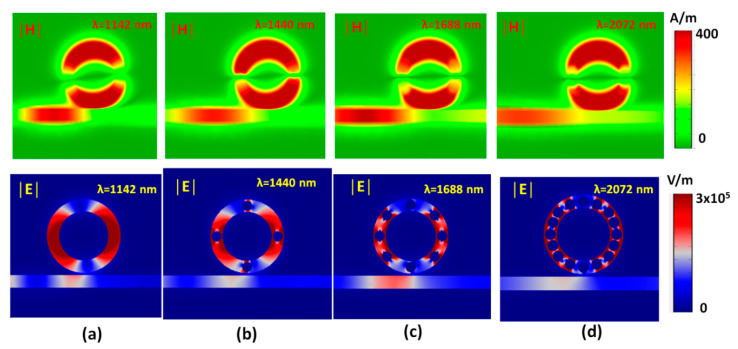
Truncate views of magnetic field intensity (|H| = (H_z_^2^)^1/2^) and electric field intensity (|E| = (E_x_^2^ + E_y_^2^)^1/2^) for the cases (**a**) without silver nanorods (at λ_res_ = 1142 nm), (**b**) with four silver nanorods (at λ_res_ = 1440 nm), (**c**) with eight silver nanorods (at λ_res_ = 1680 nm), and (**d**) with sixteen silver nanorods (at λ_res_ = 2072 nm) for mode 1, respectively.

The transmittance dips found in Figure 2 are related to the strong absorption of light in the nanoring region due to the localized surface plasmon resonance (LSPR) [55,56,57,58] and cavity plasmon resonance (CPR) [59,60,61]. Figure 3a–d depicts the magnetic field and electric field intensities for the cases without silver nanorods (at λ_res_ = 1142 nm), with four silver nanorods (at λ_res_ = 1440 nm), with eight silver nanorods (at λ_res_ = 1680 nm), and with sixteen silver nanorods (at λ_res_ = 2072 nm) of mode 1, respectively. It is evident from Figure 3 that the confinement light at the corresponding λ_res_, i.e., SPPs are confirmed at the left side of the straight waveguide and the nanoring. The nanoring can be regarded as a Fabry-Perot cavity. The SPP is coupled to the nanoring well at λ_res_, and the transmittance of SPPs is prohibited. The SPPs are limited in the nanoring due to the destructive interference between the straight waveguide and nanoring. The |H| and |E| patterns show a standing wave profile of the cases without silver nanorods while exhibiting gap plasmon and edge plasmon enhancements on the surface of silver nanorods and nanoring of the cases with silver nanorods [62,63,64,65].

**Figure 4:**
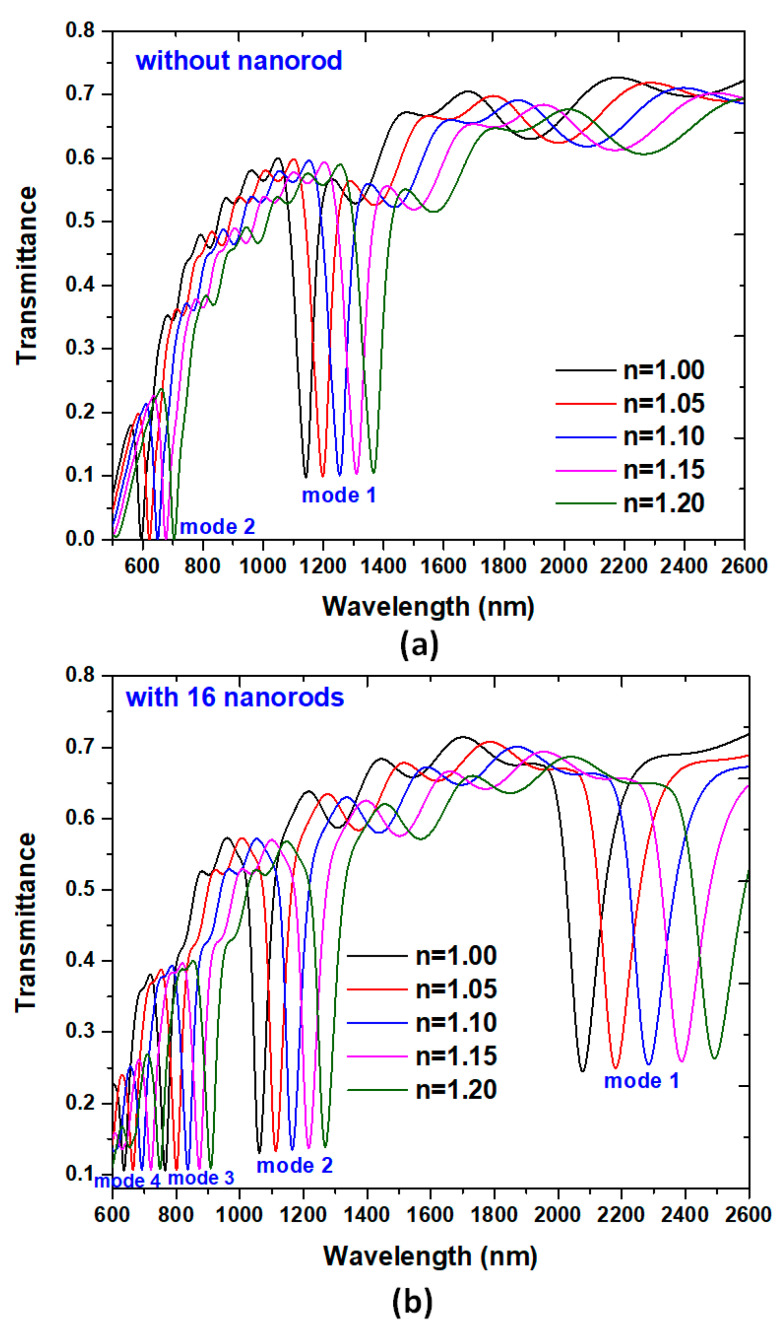
Transmittance spectra of the proposed plasmonic band-stop filter (**a**) without and (**b**) with 16 silver nanorods in the nanoring. The refractive index of the material under sensing, *n*, is assumed to be 1.0, 1.05, 1.10, 1.15, and 1.20, respectively. The other geometric parameters, *w*, *h*, *R*, and *r*, are set to be 50, 10, 100, and 20 nm, respectively.

Based on the spectral characteristics of the structures in Figure 2, we can use it as a RI sensor. Here, we choose *N* = 0 (without silver nanorods), and *N* = 16 corresponds to Figure 2 to discuss and compare the refractive index sensing applications of this device. The proposed plasmonic band-stop filter with a nanoring is sensitive to the change of the surrounding medium. The refractive index sensing characteristics can be evaluated by adding the straight waveguide and nanoring with different media. The refractive index range utilized in this work covers the refractive index of most gases. Therefore, such gases can quickly enter such a small straight waveguide and nanoring. A chamber has proposed that it can be used for this function [66]. To inspect the sensing performance, two potential factors, i.e., the S and the FOM, will be investigated to access the design’s gas sensor application. This proposed structure can be applied for designing a SPR sensor due to its straight waveguide and nanoring regions: As a result, active interaction areas with the changing media under testing. Figure 4a,b shows the transmittance spectra of the proposed plasmonic band-stop filter without and with 16 silver nanorods in the nanoring, respectively. The refractive index of the media under sensing, *n*, is set to be 1.0, 1.05, 1.10, 1.15, and 1.20, respectively. The other geometric parameters, *w*, *h*, *R*, and *r*, are set to be 50, 10, 100, and 20 nm, respectively. It is found that the results obtained from the cases with the silver nanorods (Figure 4b) are quite different from that of its counterpart without the silver nanorods (Figure 4a). When the proposed plasmonic band-stop filter is added with the different refractive index (*n* = 1.00, 1.05, 1.10, 1.15, and 1.20), the transmittance dips reveal a redshift as the increasing *n* and a linear relationship with *n* of the media under sense. It is the correlation relationship between the *n*_eff_ and the λ_res_, which come to a good agreement with Equation (1). The increase in sensitivity is due to the confinement of EM waves along the nanoring sidewall, which can widely interact with any variation in the refractive index in the nanoring [67]. In Figure 3b–d, the EM waves in the nanoring with silver nanorods can significantly enhance the excitation of SPP wave and the discontinuity of the EM waves across the interface between the straight waveguide and nanoring. It can explain the hybridization of the waveguide mode and the gap plasmon resonance mode in a manner of discontinuity of the EM waves among the silver nanorods [68]. A small refractive index variation (Δ*n*) can lead to a significant Δλ, compared to the conventional nanoring sensors (see Figure 3a).

**Figure 5:**
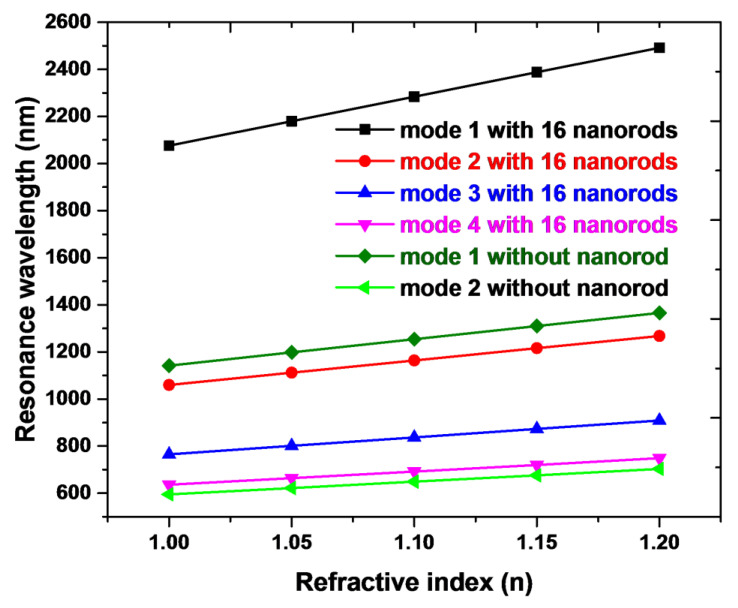
Calculated resonance wavelength (λ_res_) of mode 1–2 for the cases without silver nanorods and mode 1–4 for the cases with 16 silver nanorods versus the refractive index. The other geometric parameters, *w*, *h*, *R*, and *r*, are set to be 50, 10, 100, and 20 nm, respectively.

An excellent refractive index sensor requires having a high sensitivity (S) and a top figure of merit (FOM) [69]. Figure 5 shows the calculated λ_res_ versus *n* of the cases without and with 16 silver nanorods in the nanoring. From Figure 5, the refractive index sensitivity of the cases without silver nanorods is 1120 nm/RIU for mode 1 and 540 nm/RIU for mode 2, correspondingly. The refractive index sensitivity of the cases with 16 silver nanorods is 2080 nm/RIU for mode 1, 1040 nm/RIU for mode 2, 720 nm/RIU for mode 3, and 560 nm/RIU for mode 4, correspondingly. Note that the presence of 16 silver nanorods in the nanoring leads to an increase of structure sensitivity of 2080 nm/RIU compared to 1120 nm/RIU of the case without silver nanorods. Namely, the corresponding sensitivity can be enhanced 1.86 times (i.e., 85.71%) compared to its counterpart without silver nanorods in the nanoring. In addition, we calculate the FOM of the proposed structures. The FOM of the case without silver nanorods is 24.8/RIU for mode 1 and 21.6/RIU for mode 2, correspondingly. The FOM of the case with silver nanorods is 29.92/RIU for mode 1, 29.7/RIU for mode 2, 28.8/RIU for mode 3, and 28.0/RIU for mode 4, correspondingly. These values are more extensive than those similar structures reported in the literature (e.g., [70,71,72]) and can fit the requirement of refractive index sensing.

**Figure 6:**
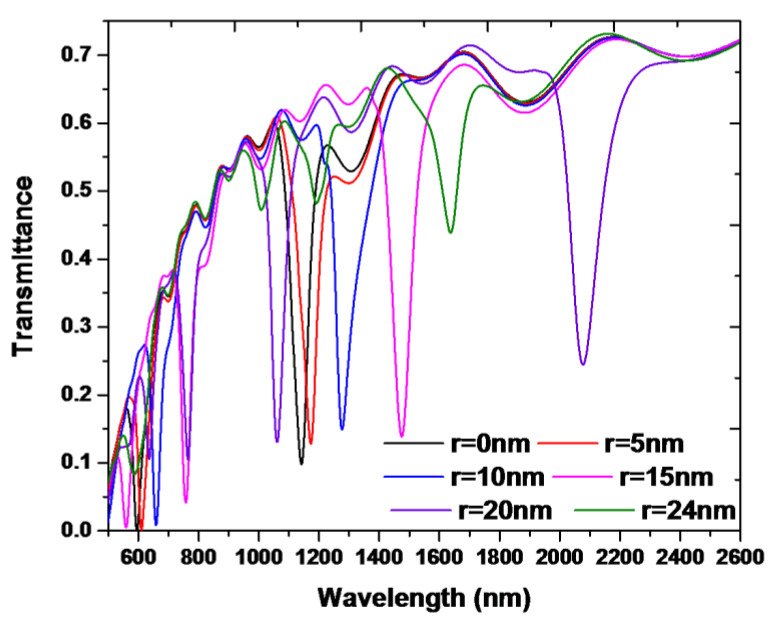
Transmittance spectra of the proposed band-stop filter with different silver nanorods radius of *r* = [0, 5, 10, 15, 20, 24] nm for the case with 16 silver nanorods in the nanoring, respectively. The other parameters are *w* = 50, *h* = 10, *R* = 100 nm, *N* = 16, and *n* = 1.0, respectively.

The resonance condition of free space in the nanoring can change by varying the silver nanorod’s radius (*r*) since the open space area between the adjacent silver nanorods has been changed, resulting in a variation of *n*_eff_ and *L* in the nanoring. It has a significant influence on the transmittance spectrum since the silver nanorods set in the nanoring located at the Bragg distance between the dielectric/metal interfaces could provide a Fabry-Pérot resonator. Figure 6 shows the transmittance spectra of the proposed plasmonic band-stop filter at a different radius of the silver nanorods with *r* = [0, 5, 10, 15, 20, 24] nm (where *r* = 0 nm stands for the absence of the silver nanorods), respectively, while the other parameters are *w* = 50, *h* = 10, *R* = 100 nm, *N* = 16, and *n* = 1.0. It can be found that r can significantly affect the transmittance dips, and the λ_res_ of the transmittance dip redshifts with the increasing of *r* when *r* is in the range of 0~20 nm. These results can be interpreted by the diverse matching impedance condition between the nanoring and straight waveguide [73]. The change of r resulted in the matching impedance when the SPP mode in the nanoring occurred. Note that the depth of transmittance dip abruptly reduces when *r* = 24 nm. It is due to the less gap plasmon resonance and cavity plasmon resonance, resulting in less coupling and the destructive interference in the nanoring. In conjunction with silver nanorods set in the nanoring, the λ_res_ of the nanoring can be tuned by changing the radius of silver nanorods without changing the nanoring structure’s outer dimension. In addition, the modulation of transmittance dips can be realized in a wide wavelength range, which shows promising applications in nanodevices.

**Figure 7:**
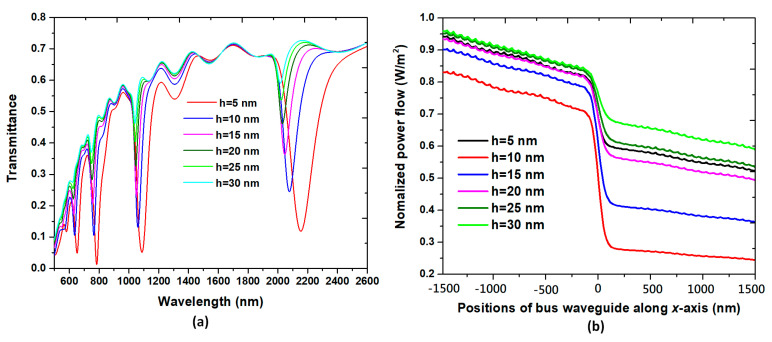
(**a**) Transmittance spectrum of the proposed plasmonic band-stop filter versus different coupling length, h. (**b**) Normalized power flow versus positions of the straight waveguide along the *x*-axis (at y = 0) in the range of −1500 to 1500 nm at different coupling length, h.

The transmittance spectrum of the proposed plasmonic band-stop filter versus different coupling length, *h*, is investigated, as shown in Figure 7a. In Figure 7a, the *h* values are varied from 5 to 30 nm in the step of 5 nm, while the other parameters are *w* = 50, *r* = 20 nm, *R* = 100 nm, *N* = 16, and *n* = 1.0, respectively. The distance between the straight waveguide and the nanoring is also a significant factor influencing the transmittance properties and the hybrid mode with confinement. It can be found that the depth of transmittance dips reduce and shift to a shorter wavelength (i.e., blueshift) with the increasing of *h*. These phenomena are attributed to the change of the coupling length (*h*) between the nanoring and straight waveguide. It indicates that the λ_res_ can be manipulated by varying the coupling length between the straight waveguide and the nanoring, which can originate from the perturbation of local SPPs mode’s *n*_eff_ in the coupling region [35]. Figure 7b shows the normalized power flow versus positions of the straight waveguide along the *x*-axis (at y = 0) in the range of −1500 to 1500 nm at different coupling length, *h*. As seen in Figure 7b, the trend of power flow decreases abruptly as the positions of *x* > 0, indicating that the nanoring containing silver nanorods can function as a plasmonic band-stop filter. The best result is for *h* = 10 nm.

**Figure 8:**
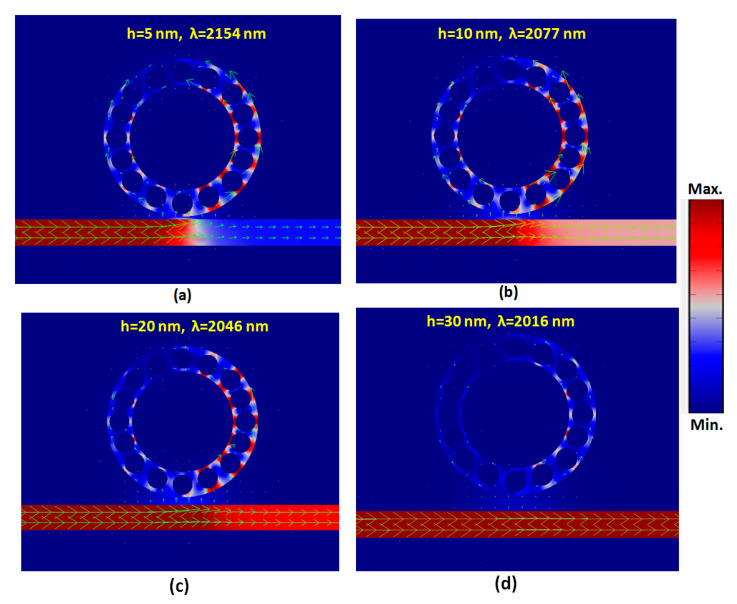
Truncate views of power flow intensities (W/m^3^) with energy arrows (green lines) for the cases of (**a**–**d**) *h* = 5, 10, 20, and 30 for mode 1, respectively.

When the incident EM wave passes through the straight waveguide, it partly couples to the SPPs mode in the nanoring, which will start to resonant in the nanoring. Different coupling lengths will result in different resonance conditions occurring in the nanoring, and the power flow intensity can examine this phenomenon existing in the straight waveguide. Figure 8a–d shows the truncate views of power flow intensities (W/m^3^) with energy arrows (green lines) for the cases of *h* = 5, 10, 20, and 30 nm of mode 1, respectively. Coupling length, *h*, plays an essential role in the coupling of photonic mode from the straight waveguide to the nanoring, as observed in these illustrations. Note that the available hybrid plasmonic waveguide mode can only form in the nanoring at a specific *h* width. It is evident from Figure 8 that the maximum power flow intensities and energy arrows can be confined near the nanometal surface, i.e., the gaps among silver nanorods and the wall of the waveguide *h* less than 20 nm. Moreover, the hybrid mode is formed in the left side of the straight waveguide when *h* = 5 and 10 nm is chosen (Figure 8a,b). As a weaker interaction in the nanoring, as seen in Figure 8c, the most energy can be conveyed to the output end of the straight waveguide, which can be attributed to interference and the coherent coupling between the mode nonradiative and mode radiative [39]. There is a weak hybrid mode formed at *h* = 30 nm, but the power flow intensity of this mode in the nanoring is too soft, which exhibits the imperfect coupling of light to the nanoring. In this case (Figure 8d), the photonic mode does not couple to the nanoring.

## 4. Conclusions

In conclusion, we numerically and theoretically investigated a highly sensitive and tunable plasmonic band-stop filter based on a MIM waveguide-coupled nanoring containing the silver nanorods by using FEM, which can be applicable for refractive index sensors. We examine the influence of geometric parameters on the transmittance spectrum and sensing performance comprehensively. It can be found that the effective refractive index can be manipulated and changed by introducing the silver nanorods in a nanoring, which can induce new SPPs modes that cannot be implemented by a regular nanoring. Results show that the sensitivity is significantly larger than that of similar structures in the literature. The proposed architecture has a significant influence on the sensitivity performance due to the presence of the silver nanorods in the nanoring. It can be found that more of the silver nanorods set in the nanoring will result in a more extensive effective refractive index and perimeter length of the nanoring. It will contribute to the increase of the λ_res_. The refractive index sensitivity can reach 2080 nm/RIU, which enhances 85.71% compared to its counterpart without silver nanorods. In addition, the maximum achieved figure of merit and quality factor reached 29.92 and 29.67, respectively. In comparison with other similar designs, the more compact dimension and ease of tunability are the most prominent merits of the proposed plasmonic sensor.
